# Thermoregulatory Responses of Heat Acclimatized Buffaloes to Simulated Heat Waves

**DOI:** 10.3390/ani10050756

**Published:** 2020-04-26

**Authors:** Alfredo M. F. Pereira, Reíssa A. Vilela, Cristiane G. Titto, Thays M. C. Leme-dos-Santos, Ana C. M. Geraldo, Júlio C. C. Balieiro, Raquel F. Calviello, Eduardo H. Birgel Junior, Evaldo A. L. Titto

**Affiliations:** 1 Mediterranean Institute for Agriculture, Environment and Development, Institute for Advanced Studies and Research, Universidade de Évora, Pólo da Mitra, Ap. 94, 7006-554 Évora, Portugal; 2 Laboratório de Biometeorologia e Etologia, FZEA-USP, Faculdade de Zootecnia e Engenharia de Alimentos, Universidade de São Paulo, Pirassununga SP 13635-900, Brasil; 3 Departamento de Nutrição e Produção Animal, Faculdade de Medicina Veterinária e Zootecnia, Universidade de São Paulo, Pirassununga SP 13635-900, Brasil; 4 Departamento de Medicina Veterinária, FZEA-USP, Faculdade de Zootecnia e Engenharia de Alimentos, Universidade de São Paulo, Pirassununga SP 13635-900, Brasil

**Keywords:** Buffalo, heat wave, acclimatization, heat stress

## Abstract

**Simple Summary:**

The simulated heat wave triggered a relevant thermoregulatory response in heat-acclimatized buffaloes. During the simulated heat wave the high respiratory rate and seating rate limited the heat storage and the levels of hyperthermia. Moderate and transient hyperthermia combined with complete recovery of homeothermy during the night prevented changes in blood parameters, except for ion potassium. The high sweating rates seem to have been crucial for the homeothermy’s maintenance. For the first time, early in the morning, adaptive hypothermia was recorded in buffaloes.

**Abstract:**

Climate change is seen as a significant threat to the sustainability of livestock production systems in many parts of the world, particularly in tropical regions. Extreme meteorological events can result in catastrophic production and death of livestock. Heat waves in particular can push vulnerable animals beyond their survival threshold limits. However, there is little information about buffalo responses to sudden changes in the thermal environment, specifically the heat waves. This study aimed to quantify the thermoregulatory and blood biochemical responses of heat-acclimatized buffaloes to a simulated heat wave. The experiment was designed in a climatic chamber with two periods of 4 days each. Twelve heat acclimated buffalo heifers aged 18 months were used. The climatic chamber environment was set as follows: 4-day period (P1) simulating the same weather conditions of a summer in humid tropical climate used as a baseline, with daily cycle with Ta and RH at 27 ± 1 °C and 76% from 0600 h to 1900 h and 24 ± 1 °C and 80% from 1900 h to 0600 h, and 4-day period (P2), simulating a daily heat wave cycle, from 0600 h to 1900 h with Ta and RH kept at 36 °C and 78% and from 1900 h to 0600 h, 27 °C and 74%. All animals were subject to both treatments and data were analyzed by a repeated measure analysis of variance, with post-hoc pooling comparison performed by Tukey’s test. In P2, there was observed a significant increase in respiratory frequency (*p <* 0.01), found four times in P1. The sweating rates were quite high in both periods; still, there were significant increases in P2 compared to P1 (*p <* 0.01) (4931 and 3201 g/m^2^/h, respectively). A slight but significant increase in rectal temperature was observed during the day (*p <* 0.01), with a rising until 1900 h. The simulated heat wave in P2 did not affect the values of the erythrogram or leukogram, excluding the significant reduction in K^+^ (*p <* 0.05). The low heat storage and the subsequent fast and full recovery of the thermal balance late afternoon appear to be related to the high sweating rate values. The massive sweating rate emphasizes its relevance in the maintenance of buffalo homeothermy. The absence of changes in hematological parameters has revealed the considerable physiological resilience of buffaloes toward simulated heat waves.

## 1. Introduction

Climate change is seen as a significant threat to the sustainability of livestock production systems in many parts of the world, particularly in tropical regions. Extreme meteorological events can result in catastrophic production and death of livestock. Heat waves in particular can push vulnerable animals beyond their survival threshold limits, as reported by numerous studies [1,2,3,4,5]. A heat wave is generally defined as a prolonged period of excessively hot weather. The lack of an official definition of heat wave is based on average weather conditions in the area and on normal seasonal temperatures [6,7,8].

The water buffalo is well-adapted to humid tropical climates. However, its geographical dispersion suggests its remarkable adaptability to different regions and climates. Even so, when exposed to high temperatures it can trigger several thermoregulatory responses together with decrease in food intake and efficiency and utilization of nutrients, as well disturbances in water metabolism, protein, energy and the ions balances, hormonal secretions, enzymatic reactions and blood metabolite levels [9]. There is however little information about buffalo responses to sudden changes in the thermal environment, specifically heat waves. 

The intensity of the thermoregulatory responses of cattle and buffalo to heat stress are different mainly due to anatomical differences between species, such as colour and thickness of the epidermis, hair density, density of sweat glands [10,11]. Buffaloes tend to show earlier signs of significant distress than cattle when exposed to direct solar radiation. In this condition, the buffalo’s body temperature, respiratory and pulse rate increase earlier and faster than those of cattle [12,13]. By contrast, the rectal temperature of buffaloes decreases quicker when they are moved into the shade [14] or sprayed with water [15] mainly due to the high density of the skin’s blood vessels that promotes faster heat loss by conduction, radiation and convection [11], despite the lower density of sweat glands. However, many of these responses refer to acute stimulus, in which animals rapidly change from thermoneutrality to heat stress without prior conditioning [16]. However, when animals are acclimated to heat, there is higher evaporative heat loss together with more stable body temperature [17,18].

The responses to the sudden change in the thermal environment highlight the adaptive capacity of animals and are the basis for the early acclimatization [19]. When a prolonged period of heat stress occurs, buffaloes try to acclimatize, which involves phenotypic responses, reflected in adjustments in hormonal concentration and modifications in target tissue responsiveness to hormonal stimuli [3,20,21]. In case of persistent and relevant heat storages along with circadian cycles, this triggers faster acclimatization, especially when heat stored during the day is not fully dissipated through night-time [22,23]. Acclimation to heat is indicated by a trend to a normal body temperature circadian cycle and by the almost return of regular feed intake [24,25].

However, even acclimatized to heat, an abnormal and rapid change in thermal environment, such a heat wave, requires the activation of additional emergency physiological responses to avoid sudden death [26,27]. Some studies are reporting thermoregulatory responses in heat-acclimatized animals [28,29]. However, regarding buffaloes, few studies have been carried out on acclimatization and its physiological consequences [20,30].

This study aimed to quantify the thermoregulatory and blood biochemical responses of heat-acclimatized buffaloes to a simulated heat wave.

## 2. Material and Methods

### 2.1. Animals and Experimental Conditions

The experiment occurred during the peak of the summer season, at the campus of the Faculty of Zootechny and Food Engineering, University of São Paulo, Pirassununga, Brazil. Twelve nonpregnant Mediterranean buffaloes aged 18 months with an average body weight of 380 kg were used. All buffaloes belonged to the same herd and were full acclimatized to the tropical summer conditions.

The study was carried out in a climatic chamber of 45 m^2^, enabling the simultaneous housing of 6 animals. Air temperature control was achieved by heating or chilling the airflow, entering the climatic chamber through several openings, placed at the height of 2.1 m, along with its longitudinal axis. The environmental parameters inside the chamber were set through the central control, placed outside the climatic chamber. The temperature and humidity control system responded with reasonable speed and accuracy to the elicited thermal changes and showed initially low thermal inertia.

Due to the limited space of the climatic chamber, the experiment took place in two different phases with six buffaloes each.

All buffaloes were handled, head haltered and trained for one and a half months before the experiment, with the aim of decreasing reactivity and for better adjustment to the new handling and environment. Before the experiment began, the buffaloes went to the climatic chamber for a short time for habituation to noise, scent and space. Only when full desensitization of the buffalo to the chamber environment and handling procedures were observed did the adaptation period begin, with the placement of the animals in their final places. At the beginning of the experiment, all buffaloes were very calm and presented low reactivity to people and handling procedures. The buffaloes were kept in individual stalls (2.5 × 1.4 m) and restrained by a head halter. Diet was composed of concentrate and maize silage (proportion of 20/80 on a dry matter basis) with 76% Total Digestible Nutrients (TDN) and 18% Crude Protein (CP). Food, water and mineral mixture were available ad libitum, with fresh food provided twice per day (0600 h and 1600 h). The buffaloes were kept in individual stalls (2.5 × 1.4 m) and restrained by a head halter. Diet was composed of concentrate and maize silage (proportion of 20/80 on a dry matter basis) with 76% TDN and 18% CP.

### 2.2. Experimental Design

Each trial lasted 12 days. There was a 2–day adaptation period for the buffaloes’ adjustments or habituation to the various routines they would face during the test and the ten days for data collecting. The data were obtained in two periods for a better understanding of the animals’ response to heat waves. Period 1 values serve as the baseline for each buffalo and constitute the individual comparison for the simulated heat wave responses. During Period 1 (P1), the environmental condition was set to be similar to those observed outside of the climatic chamber, i.e., such as those found naturally in a tropical climate summer; whereas Period 2 (P2) was set to a simulated heat wave. The climatic chamber environment was set as follows: (P1) 4 days, with air temperature (Ta) and relative humidity (RH) daily cycle with Ta and RH at 27 ± 1 °C and 76% from 0600 h to 1900 h hand 24 ± 1 °C and 80% from 1900 h to 0600 h; (P2) 4 days, simulating a daily cycle of heat wave temperatures, with Ta and RH kept at 35 ± 1 °C and 76% from 0600 h to 1900 h and 27 ± 1 °C and 80% from 1900 to 0600. Two data loggers were used to measure the air temperature and relative humidity both periods (HOBO U12 Temp/RH/Light/External Data Logger, Onset Computer Corporation, Bourne, MA, USA). The data loggers were placed in two locations at a height of about 2 m above the floor. The temperature–humidity indices (THI) [31] were calculated as follows:

(1) $$THI=\left\{1,8.Ta-\left[\frac{1-rH}{100}\right]\times \left(Ta-14,3\right)+32\right\}$$

On the fifth day of each period, blood samples were collected at 1400 h, and no other measurements were taken this day. The blood samples were collected in the same order in both periods.

### 2.3. Physiological Variables

Variables related to thermostability and sensible and evaporative heat loss were collected during the first 4 days in each period, for the evaluation of the animals’ responses to the simulated heat wave, as follows: respiratory frequency (RF) measured by observing costal movements for 60 s; rectal temperature (RT) taken using a clinical digital thermometer (Digitron, with an 8-cm flexible probe); the temperatures of the internal base of the tail (TT) and average body coat (CT), measured in five different places along the longitudinal axis of the trunk. Both were measured using an infrared thermometer Minolta Land Cyclops. All of these measurements were carried out every day at 0700 h, 1000 h, 1300 h, 1600 h, 1900 h, and 2200 h.

Sweat rate was measured daily at 1400 using a calibrated digital moisture sensor Vapometer TM (Delfin Technologies Ltd., Kuopio, Finland) that determines trans-epidermal water loss. The Vapometer uses a closed system approach, free of ambient airflow, to measure ambient relative humidity and temperature. The device is then held on the skin for 10 to 20 s until a beep identifies the end of the measurement; the value of the sweating rate appears on display in g/m^2^/h (accuracy = ± 10%). All devices were previously calibrated.

The heat storage (HS) and cumulative heat storage (CHS) were calculated by the methodology described by [32]

∆RT = (3600 × HS × A)/(Bw × cb)(2)

where ∆RT—are the differences between rectal temperatures in different hours; HS—heat storage [W/m^2^]; A—animal surface [m^2^] where A = 0.13. Bw^0.556^, (Bw—body weight in Kg), and cb—the specific heat of the animal (3.4 KJ/kg/°K).

(3) $$\mathrm{CHS}=\sum_{7}^{22}HS$$

where CHS—cumulative heat storage (W/m^2^/h); is calculated CHS—sum of the storage heat HS in different time intervals (from 0700 h to 2200 h), being considered the zero value at 0700 h.

### 2.4. Blood Parameters

Two blood samples were collected on the fifth day of P1 and P2, at 1400 from the jugular vein. One aliquot of blood was collected into the Sarstedt Monovet serum gel for the determination of sodium (Na^+^) and potassium (K^+^). The blood samples were analyzed using a Diagnostic AVL 9180 Electrolyte Autoanalyzer, Roche Diagnostics 03157334001™ with a two-point calibration performed every four hours and a one-point calibration with each test, ensuring precision and conformity.

For quantifying the constituents of erythrogram, blood samples were collected in vacuum tubes containing ethylenediamine tetraacetic acid (EDTA). The number of erythrocytes (Er) was measured using the modified Neubauer hemocytometer and diluted in the hematimetric pipette, used as a diluting solution to the fluid Gower. The packed cell volume (Ht) was performed using the microhematocrit. Hemoglobin (Hg) was quantified by the method that transforms the hemoglobin cianometemoglobin. The mean corpuscular volume (MCV), mean corpuscular hemoglobin (MCH) and mean corpuscular hemoglobin concentration (MCHC) were also quantified.

White blood cell count was performed on the total number of leukocytes (Leu) in a modified Neubauer chamber. Differential leukocyte count was conducted on blood smears prepared with fresh blood and stained with panchromatic Rosenfeld. In each blood smear, 100 leukocytes were differentiated and classified as neutrophils with a nucleus in the rod (Nrd) or with a segmented nucleus (Nsg), eosinophil (Eos), basophil (Bas), lymphocyte (Lym), and monocyte (Mon) [33]

### 2.5. Statistical Analysis

Different statistical models were used to analyse the different variables collected. All variables were tested for normality by Shapiro–Wilk test and homoscedasticity by Levene test. Considering that the results were obtained on the same animal at different times, variables RF, RT, TT, CT, HS, and AHS, were performed by a repeated measure analysis, using a model including two fixed effects, the periods (P1 and P2) and the time (0700 h, 1000 h, 1300 h, 1600 h, 1900 h, and 2200 h). Previously, sphericity was analysed by Mauchly’s test. The interaction between factors (periods and time) was performed considering the random effect of the animal as a residue. When interactions were significant, regression analysis was used to identify the response of the dependent variables as a function of time. For the variables of sweating rates, hematological parameters (Na^+^, K^+^, Er, Hg, Ht, MCV, MCH, and MCHC) and white blood cells (Leu, Mon, Lym, Eos, Bas, Nsg, and Nrd), repeated measure analyses were performed, using the Periods as a fixed effect (P1 and P2) and the random effect of animal used as residues. When a significant difference in ANOVA occurred, means were compared by post-hoc Tukey’s test. Additionally, GLM ANOVA was performed considering day within the period as a random factor, testing the occurrence of trend over the days within the periods.

All analyses were performed using the software package SPSS version 22 Copyright IBM Corp Armonk, New York, United States.

The Ethics committee certified by the following Law 11.794, of 8 October 2008, Decree 6899, of 15 July 2009, that all procedures performed in this study followed ethical standards. The animals used in the experiment had prior approval of the Ethics Faculty Committee, receiving the protocol number n°8702060420.

## 3. Results

During the experimental period, all buffaloes displayed very low reactivity toward routine procedures, and no buffalo exhibited signals of stress or any evidence that could be related to the beginning of thermoregulation failure.

**Figure 1:**
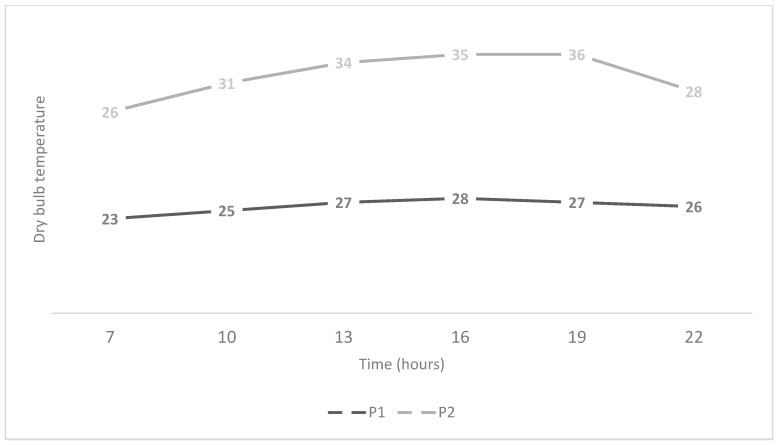
Mean values of the dry bulb temperatures observed during P1 and P2 over the day.

**Figure 2:**
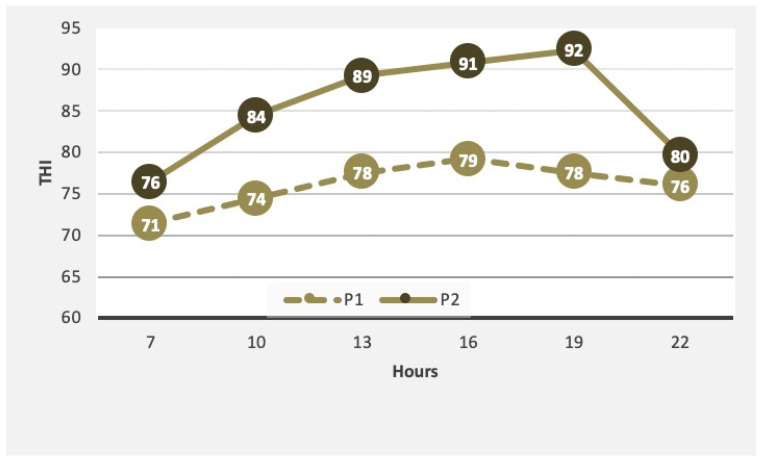
Mean values of temperature–humidity indices (THI) values observed during P1 and P2 over the day.

Figure 1 and Figure 2 show, respectively, the dry bulb temperature and the THI mean values over the course of hours in P1 and P2.

There were no significant differences in the mean values of the physiological variables among the days in each period. For this reason, the values observed on each day are analyzed separately, but only the mean values.

### 3.1. Coat and Epidermis Temperatures

The means of tail temperature (TT) and coat temperature (CT) showed similar trends (Table 1). There were significant differences (*p <* 0.01) between periods and among hours. The values of TT and CT obtained in P2 were significantly higher than P1 (*p <* 0.01). The higher values of TT reveal intense peripheral vasodilatation, while CT values in P2 show the combined effect of higher TT driving to the warming of the coat with a low thermal gradient to the air chamber’s temperature, which contributed to a low sensible heat loss by convection and radiation.

The patterns of CT and TT over the hours were very similar, both exhibiting significant differences along the hours (*p <* 0.01). In P1 the highest value of CT (34.28 °C) and TT (35.13 °C) were observed at 16:00 h, while in P2 the maximum values were reached at 1900 h, 38.94 °C and 37.96 °C respectively for CT and TT. In P2, at 2200 h, the TT remains significantly higher than in P1. This difference seems to indicate persistent peripheral vasodilatation, indicating an additional need for sensible heat loss, due to the more favorable thermal gradient.

### 3.2. Respiratory Frequency

There were significant differences in respiratory frequency (RF) means among periods and hours (*p <* 0.01) (Table 1). In P1, the values were quite low, and there were no significant differences among the hours (*p* > 0.05), which normally indicates an irrelevant effort to lose evaporative heat by respiration. However, during P2, the means of RF increased nearly four times the values observed in P1. The RF was rising significantly (*p <* 0.01) from 0700 h (26 mov/min) until reaching a maximum value at 1900 h (87 mov/min). After the decreasing of the chamber temperature at 1900 h, there was a significant RF reduction, revealing a lower demand for losing heat by thermal polypnea.

### 3.3. Sweating Rate

**Figure 3:**
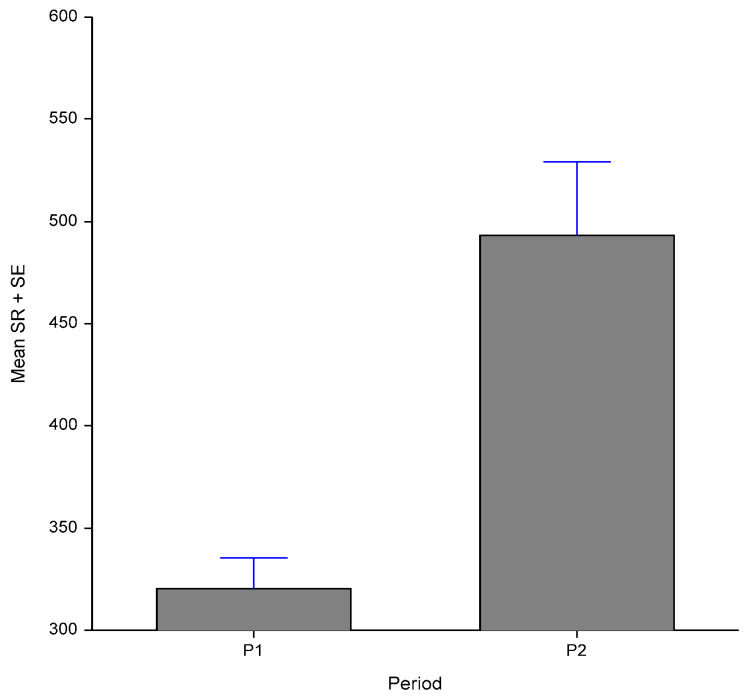
Mean values and standard error of means of sweating rates in P1 and P2.

The sweating rate was considerably high in both periods, contrasting with that which occurred with the respiratory rate. The high sweating rates observed in P1 (320.18 g/m^2^/h), reveal the need to increase the evaporative heat loss due to the THI values in P1. Although, the mean values obtained in P2 were significantly higher (493.13 g/m^2^/h) than those observed in P1 (*p <* 0.01), in P2 the sweating rates dispersion means were much higher than those observed in P1 (Figure 3), which shows some significant individual differences in sweat production under high-temperature environmental conditions.

### 3.4. Rectal Temperature, Heat Storage, and Cumulative Heat Storage

There were significant differences in rectal temperature (RT) mean values between periods and among hours (*p <* 0.01) (Table 1). During P1, there were only slight but significant (*p <* 0.01) variations in RT along the day. The RT had been increasing since 0700 h, reaching maximum values at 2200 h (38.88 °C). However, in P2, the RT mean values were significantly higher (*p <* 0.01) than those observed in P1. Only until 10:00 h were the mean values of RT similar in both Periods (*p >* 0.05). The differences between the Periods increased throughout the day, with the highest difference reached at 1900 h (0.89 °C). Moreover, in P2, there was an outstanding reduction of RT after 1900 h, as well as the surprisingly low RT at 0700 h (*p <* 0.01).

The calculated values of heat storage (HS) were significantly higher in P2 than in P1, showing similar trends as the RT. At P1, there was a marked increase in heat storage between 1600 h and 1900 h. This situation did not occur in P2, where the peak was reached at 1300 h.

**Figure 4:**
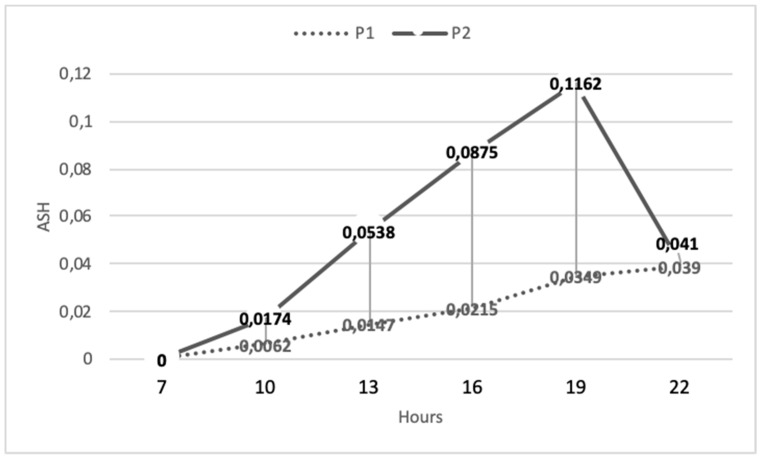
Mean values of the cumulative heat storage in P1 and P2.

The cumulative heat storage (CHS) patterns were different between P1 and P2 (Figure 4). In P2, the total heat storage was triple at 1900 h than that registered in P1. However, in P2, after the decrease of the chamber’s temperature, the remarkable heat loss presented by the animals provided a reduction in the cumulative stored heat, which ended with similar values of CHS in both periods at 2200 h.

### 3.5. Hematological White Blood Cells and Electrolytic Variables

Between P1 and P2, there were no significant differences in the mean values of any hematological variables (Table 2), except for the mean values of K^+^ that were significantly lower in P2 (*p <* 0.01) (4.4 and 3.9) despite the absence of differences in the mean values of Na^+^.

Likewise, hematological parameters of the white blood cells did not show any differences in the mean values of white blood cells between periods (Table 3). The Nsg and Nrd showed slightly higher tendency values in P2, but the differences were not significant, probably due to the individual differences observed. A similar tendency has occurred with Bas, yet, with higher values registered in P1.

## 4. Discussion

Heat waves trigger relevant adaptive challenges. The sudden increase in temperature reduces the possibility of sensible heat loss and implies an intense latent heat loss. If this response is not fast and capable enough, then there is rapid heat storage and prolonged periods of hyperthermia. The physiologic processes for coping with heat stress include previously sizeable peripheral vasodilation with increased blood flow to the skin surface, followed by a more profuse sweating and later a higher respiratory frequency [3,16,30,34].

Acclimatization to heat implies theoretically an increased ability to overcome the challenges caused by sudden changes in ambient temperature. Such responses are expressed by more evaporative heat loss, lower heat storage and more attenuated biochemical and endocrine thermal stress responses [28]. Studies highlighting buffalo responses to heat waves are limited. A few studies relate the reactions of buffalos under conditions of acute heat stress, but these do not show the circumstances under which the animals were kept. Possibly due to different acclimatization conditions, the buffaloes’ responses to acute stress show high heterogeneity.

### 4.1. Coat, Epidermis Temperatures and Sensible Heat Loss

In the absence of solar radiation, the skin temperature is more strictly related to the level of peripheral vasodilatation and consequently with the sensible heat exchange with the environment. The animal’s skin temperatures, together with the ambient temperature, allow for inferring about the level of heat exchange by convection and radiation.

The sensible heat loss by radiation and convection tends to be high in buffaloes, because of extensive blood vessels in the skin, with the arteries branched more frequently giving rise to many arterioles and capillaries [11,12]. The buffaloes present a vast ability to lose sensible heat. The extensive network of the peripheral blood vessel, the sparse coat, and the dark skin enhance the heat loss by convection and radiation [11].

**Figure 5:**
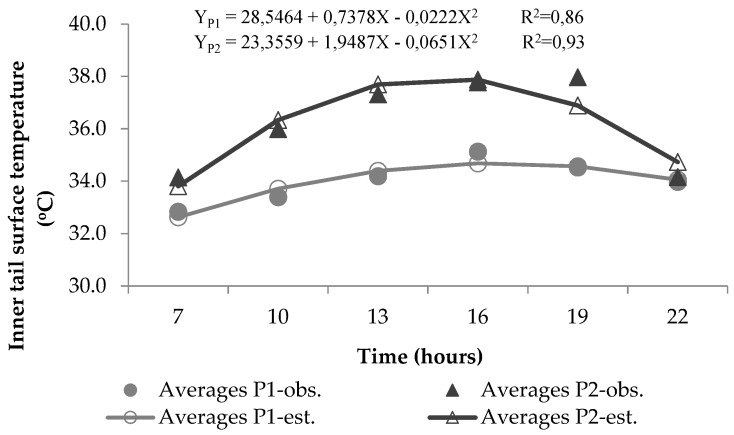
Regression equations between the variable tail temperature (TT) along the hours in P1 and P2 (observed values vs. predicted values).

In P1, CT and TT showed similar patterns throughout the day, showing smaller amplitudes. At P2, the temperature during the day was quite the same as the core temperature of the animals, limiting the sensible heat exchanges. However, in P2, the patterns changed both in the amplitudes throughout the day, and at the time they were reached. TT reached the maximum at 1900 h (37.96 °C), shown in the regression equation (Figure 5). For both periods, the best fit is a quadratic regression, although in P2 the higher residues were at 1900 h, and penalize the curve adjustment. Haque [35] reported a close positive correlation between heat conductance with both mean skin and body temperatures.

Despite the extensive peripheral vasodilation, evidenced by the high TT, the thermal gradient between animal and the ambient temperature does not favor heat loss by convection and radiation throughout the day. This emphasizes the importance of evaporative heat loss. The maintenance of high TT at 1900 h shows thermoregulatory effort to maximize the sensible heat loss. However, after the decreasing of the chamber temperature, the new thermal gradient (about 6 °C) anticipates a significant sensible heat loss throughout the night period. This sensible heat loss contributed to an RT at 0700 h that was lower than that which occurred at the same time in P1.

### 4.2. Respiratory Frequency, Sweating Rate and Evaporative Heat Loss

Simulated heat waves (P2) led to significant increases in evaporative heat loss. In P1, the evaporative heat loss pathways, panting and sweating, showed distinct performance. Under the mild environmental temperatures of P1, the sweating rates, notably high, were the primary thermoregulatory response of the buffaloes. The better thermal gradient provides a significant sensible heat loss, which, together with high sweating rates, were enough to prevent the onset of the polypnea and the maintenance of low heat storage. The respiratory rate always remained low and stable throughout the day. Usually, the absence of a growth pattern in respiratory frequency during the day is associated with thermoneutrality [31,36]. However, the remarkable high sweating rates in P1 suggest the necessity to dissipate heat. Although sweating may occur in the absence of a relevant increase in core temperature, a high skin temperature, per se, cannot elicit full-scale sweating rate, without a simultaneous central nervous system facilitation, based on the rising tendency of the core temperature [9]. Besides, these high rates of sweating could reveal that buffaloes are in fact heat acclimatized, with low thresholds of sweat gland’s responses with a high sweat production [11,37].

The simulated heat wave, (P2) provided marked thermoregulatory responses in both evaporative heat loss pathways but did not prevent rises in body temperature.

All buffaloes used both evaporative heat loss pathway more intensely and for a longer time. The RF increased through the day, with maximum values of 87 mov/min at 1900 h, which are like those observed by [35] but beyond the values referred by [38]. This result is similar to other studies in continuous or cycling heat exposure [22,39,40], which correlated high RF with low thermal tolerance.

The RF has been widely used as an indicator of heat stress in cattle and buffaloes [38,41,42]. The increase in RF is an essential thermoregulatory response to maintain homeothermy under a condition when sensible heat loss is unavailable. In cattle, the heat loss from respiratory tract accounts for approximately 15% of the total heat dissipation under heat load [41].

Jawahar (2008), in a climatic chamber, found a significant increase in respiratory rates in buffalo, after exposure to a temperature of 40 °C for 4 h. Other studies with buffaloes reveal that in exposure to hot and arid conditions at a temperature of 44.1 °C, respiration rate increased by 5 ± 6 times, showing the buffaloes with their tongue protruded and experiencing severe sialorrhea [43].

**Figure 6:**
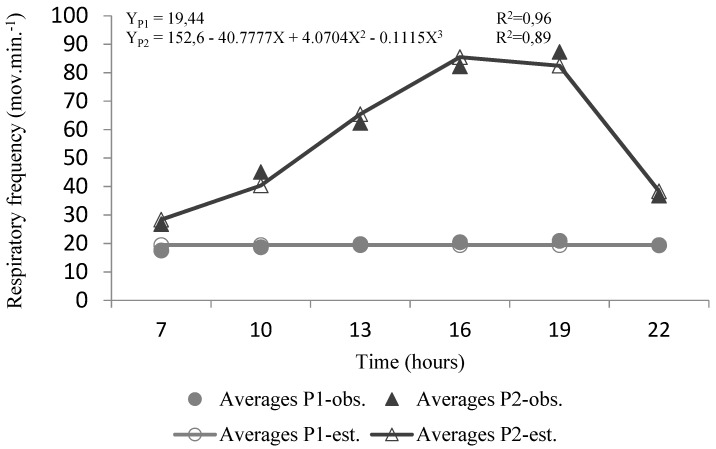
Regression equations between the variable of respiratory frequency (RF) over the hours in P1 and P2 (observed values vs. predicted values)**.**

The slowing of the respiratory rate observed after 1900 h suggests less need to lose heat together with better conditions to lose more heat by convection and radiation. Chaiyabutr et al. [44] reported that at higher than 30 °C, the rectal temperature tends to increase to 0.0033 °C/min while the respiration rate rises rapidly to about three to four times the typical values. In our study, the RF has shown a significant increase and then a marked decline from 1900 h, represented by a polynomial equation of the third degree (Figure 6). However, the noticeable increase in RF observed in P2 was not sufficient to prevent slight hyperthermia. Chaiyabutr [44] reported that at higher than 30 °C, the rectal temperature tends to increase to 0.0033 °C/min while the respiration rate rises rapidly to about three to four times the basal values.

Sweating rates has also increased significantly in P2, despite the already high values observed in P1. Even in P1, those sweating rates are higher than those observed in other studies. Titto [38] in the climatic chamber obtained an increase in the buffalo sweating rate from 107.3 to 252.2 g/m^2^/h when the temperature was raised from 28.2 °C to 34.7 °C. Similar results were observed by Joshi [12].

Several studies have reported low sweating rates in buffaloes [12], whereas other studies, with buffaloes under direct solar radiation, present higher sweating rates such as 463 g/m^2^/h [45], much more similar than those observed in P2. Assuming the sweat gland surface per unit of skin area in cattle is at least three times that of buffaloes [12], it seems that under thermal heat conditions the sweat glands of buffaloes are nearly as functional as those of cattle after being exposed to hot conditions [45]. Our results also suggest that summer acclimatized buffaloes have about the same threshold level for stimulus by thermal stress as cattle, this may be related to preconditioning of the sweat glands to summer acclimatization, which is confirmed by the high sweating rate values observed in P1.

### 4.3. Rectal Temperature and Heat Storage

The most commonly used variable to assess heat stress is RT because it is easy to measure, well documented in the literature, and the heritability is moderately low [46].

All farm animal species exhibit a daily variation of body temperature, typically in the form of a monophasic rhythm which can be altered by environmental stressors (e.g., adverse air temperature or weather events).

In P1, RTs remained quite stable during the day, although show a small increase until 2200 h (*p* > 0.05). While mean data provides a measure of the response of animals to thermal stress, dynamic based data collected frequently better represents the coping capabilities.

Several experiments carried out to compare thermoregulation in buffaloes with that of temperate cattle revealed that the rectal temperature of buffaloes presents similar patterns to that of cattle [14,15,17].

The observed RT in this study were slightly higher than those observed in other studies, which could indicate that buffaloes are not under thermoneutrality. Under thermoneutral conditions, the authors of [21] found that the lowest rectal temperature observed was 37.9 °C in Tharparkar and 37.8 °C in Karan Fries heifers at 0700 h, which increased up to the highest values of 38.7 ° C in Tharparkar and 38.8 °C in Karan Fries at 1500 h.

Thus, RT, as well HS, seem to confirm that the animals were in conditions of mild thermal stress, combining small heat storage with high sweating rates. An addition of the mild heat stress seems to be the increase of the rectal temperature even after the decrease of the ambient temperature. This time lag tends to occur when there are better conditions to lose sensitive heat and less activity of the sweat glands [47,48]. Koga [49], comparing the thermoregulation of buffaloes in the climatic chamber, showed that the rectal temperature of buffaloes varied according to the diurnal changes in air temperature.

In adult buffaloes, the increase in ambient temperature resulted in a rise of RT from 37.8 °C to 39.0 °C in heifers and from 37.9 °C to 39.7 °C in lactating buffaloes. Core temperature and respiration frequency are commonly considered the most sensitive indicators of heat stress among the physiological reactions [46].

**Figure 7:**
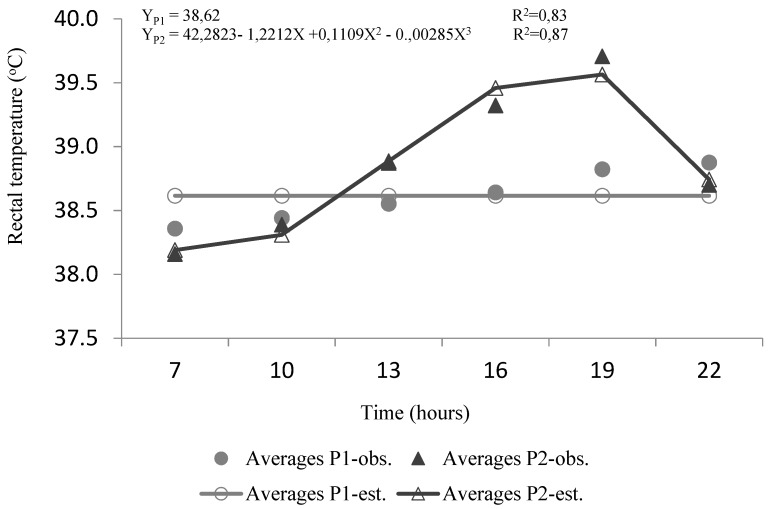
Regression equations between the variable RT over the hours in P1 and P2 (observed values vs. predicted values).

In the current study, when buffaloes were exposed to the simulated heat wave period (P2), the RT showed a pattern that reflects some incapacity to maintain homeothermy. This pattern resembles those observed in several studies when buffaloes were under heat stress and are the result of impairment of heat loss capacity [9,43]. Krishnan [50] in chamber studies also reported a significant increase in rectal temperature, respiration rate and pulse rate in heat-stressed buffalo calves when compared to control animals. However, in this study, the RT only presented a modest increase through the day (Figure 7) and also displays a sudden decrease in RT after 1900h. These limited rises in RT were mostly due to the high intensity of evaporative heat loss. These typical thermoregulatory responses, when left unchecked during a severe heat wave, with excessive heat loads, can lead to a pathological state resulting in impaired performance or even death [31]. Body temperature and heat storage patterns were influenced by the increasing temperature of the climatic chamber until 1900 h, as well as the absence of solar radiation. Under solar radiation conditions, the radiant temperature often reaches maximum values during the morning, and the animals tend to show earlier, and eventually more significant, heat storage [47].

The low thermal inertia observed after 1900 h may be the combined effect of the maintenance of persistent high sweating rate with increased sensible heat loss, due to the most favorable thermal gradient. Several authors emphasize that animals heat acclimatized show a fast return to normal body temperature rhythms when environmental temperature decreases [24,33].

It is essential to highlight that the mean RT values observed in P2 at 0700 h were significantly lower (*p* < 0.05) than those observed in P1 at the same hour. This unlikely adaptation has never been reported in buffaloes. This transient hypothermia is quite common in animals that live in the arid environment. The heat storage during the day is lost during the night-time period mainly by radiation and convection, taking advantage of the high thermal gradient overnight, which allows them to have a higher heat storage during the day, delaying the loss of water by evaporative cooling [51].

### 4.4. Hemogram, Leukogram and Electrolytes

The mean values of hemogram and leukogram did not differ between Periods. The hematological parameters were similar to others obtained under thermoneutrality [52,53,54]. However, these results are not in agreement with other studies that reported a decrease of the hemoglobin and hematocrit in severe heat stress [55]. Silva [56] reported that heat stress in buffaloes could influence hematological values, increasing the hemoglobin, red blood cells and leukocytes.

An increase in PCV, erythrocyte count (RBC) and hemoglobin concentration (Hb) may occur during dehydration or splenic contraction induced by sympathetic nerve activation or circulating catecholamines [57].

The hematocrit, volume of extracellular fluid, blood and plasma can change between seasons [49,58], which probably affects the thermoregulatory responses of these animals. The decrease in hematocrit is due to the combined effect of the decreased hematopoiesis and hemodilution, as a consequence of the excessive water consumption [22,34]. As the ambient temperature increased, blood volume increased considerably (from 47.9 to 55.8 mL/kg), while there was no change in extracellular fluid. Moreover, acute heat stress also promotes an increase in plasma volume and blood, as well as a decrease in packed cell volume [59].

Several studies have pointed out the relationships between heat stress and immune responses. Some authors showed an impairment and others no effects of high environmental temperatures [52,60,61].

In the current study, the values of leukogram follow the same tendency as hemogram, with no differences between Periods. The values observed in both periods are quite similar to those described in thermoneutrality [52,62]. The leukocyte counts in P2 heat contradict the results obtained in several studies, where there was an increase in the number of leukocytes such as eosinopenia and lymphopenia [63].

Depending on heat adaptation, different breeds may vary in their leucocytic response to heat stress. Marwari sheep breed which have better adaptability, demonstrated by an increase in lymphocyte count but a decrease in neutrophil count, whereas Chakla sheep breed have lower adaptability and showed a decrease in lymphocyte count but an increase in neutrophil count [63,64].

Nevertheless, the wide variety of experimental conditions regarding breeds, severity and length of heat stress, recovery opportunities, as well the specific immune functions taken into consideration, may explain the result’s discrepancy [65,66]. The absence of negative consequences on the immune system could be a consequence of the moderate and transient hyperthermia with a complete night-time recovery.

Heat stress often causes changes in the electrolyte balance, because there is a decrease in mineral intake followed by a reduction in their retention, and at the same time, the total excretion of all minerals increases significantly [16]. Fetman [67] discusses that acute heat stress causes changes in electrolyte balance, due to the abnormal water and electrolyte losses by sweating, higher water consumption, and changes in the rate of excretion. The heat stress especially affects the Na^+^, K^+^ and Cl^-^. The P2 conditions did not influence the concentrations of Na^+^ but caused a significant decrease in K^+^. Ruminants produce sweat that is high in K^+^ and lower in Na+ concentrations. The increased sweating rate does not interfere with the plasma concentration of Na^+^. Even if moderate isotonic dehydration occurs, the Na+ and Cl- concentrations remain unchanged, with proportional losses of water and solutes [65].

In P2, the decrease in K^+^ is consistent with the literature. In this study, the massive sweating rate observed can justify the higher losses in K^+^ in association with the eventual increased urinary excretion. Beede [68] reported, under subtropical climate, that the K^+^ loss from the skin increased five times during peak heat stress. Chaiyabutr [59] shows that the reduction in K^+^ often matches the increase in water turnover and the concentration of glucose.

Singh and Newton [28] reported an increase in sweating rates during hyperthermia and a correspondent loss of K^+^. Furthermore, with the exposure to acute heat stress, it is quite common that buffaloes show signs of distress with rapid shallow breathing, which usually tend to produce transient alkalosis. The intense polypnea observed in P2 can shift K^+^ into cells in exchange for H+ release from intracellular buffers. These actions probably could also lead to decreased K^+^ levels in the plasma during P2.

## 5. Conclusions

The thermal conditions imposed by the simulated heat waves (P2) had significant physiological responses, compared to the scenario observed in mild heat stress (P1). In P2, both respiratory frequencies and sweating rates were very high, in particular, the sweating rates are far beyond the more common values reported. Nevertheless, this large evaporative heat loss did not prevent a slight but significant increase in RT over the day. The overnight thermal gradients and the persistent sweating rate allowed for a rapid heat loss of the heat stored during the day. This night-time recovery is imperative for survival for ruminants when severe heat challenges occur.

The negative balance in the K^+^ could be related to the high sweating rate, which has not been compensated for enough in the short term. Although several studies highlight the importance of bathing in cooling buffalo, our findings suggest the essential role of sweating rate in the maintenance of buffaloes’ homeothermy, mainly when the shade is the only resource available. The simulated heat waves in acclimatized buffaloes reveal exuberant evaporative heat loss resulting in minor changes in heat storage. However, the absence of responses in the blood parameters seems to indicate considerable physiological resilience to heat waves.

## Figures and Tables

**Table 1:** Means of the physiological variables observed during P1 and P2 over the hours.

Variables	Treatments	Hours					
		7	10	13	16	19	22
CT (°C)	P1	30.22 ^dB^	31.49 ^cB^	33.22 ^bB^	34.28 ^aB^	33.53 ^abB^	32.92 ^bB^
	P2	33.20 ^cA^	37.32 ^bA^	38.66 ^aA^	38.78 ^bA^	38.94 ^aA^	33.81^cA^
TT (°C)	P1	32.83 ^dB^	33.39 ^cdB^	34.19 ^bcB^	35.13 ^aB^	34.52 ^abB^	33.98 ^bcB^
	P2	34.14 ^cA^	35.99 ^bA^	37.31^aA^	37.77 ^aA^	37.96 ^aA^	34.15 ^cA^
RF (mov/min)	P1	17.56 ^aB^	18.67 ^aB^	19.78 ^aB^	20.44 ^aB^	20.92 ^aB^	19.33 ^aB^
	P2	26.78 ^eA^	45.11 ^cA^	62.33 ^bA^	82.22 ^aA^	87.33 ^aA^	36.78 ^dA^
RT (°C)	P1	38.36 ^dA^	38.44 ^cdA^	38.55 ^bcB^	38.64 ^bB^	38.82 ^aB^	38.88 ^aA^
	P2	38.16 ^fB^	38.39 ^eA^	38.87 ^cA^	39.32 ^bA^	39.71 ^aA^	38.70 ^dAt^
HS (W/m^2^/h)	P1	0.00 ^cA^	0.0021 ^bcB^	0.0028 ^abB^	0.0023 ^abcB^	0.0045 ^aB^	0.0014 ^bcA^
	P2	0.00 ^dA^	0.0058 ^cA^	0.012 ^aA^	0.0112 ^abA^	0.0096 ^bA^	−0.0251 ^eB^

In each column, variable mean values with different capital superscript letters are significantly different between periods (*p* < 0.05). In each row, different small superscript letters in the same line are different among hours.

**Table 2:** Means of hematological and electrolytic variables in P1 and P2.

Treatments	Variables						
	Er $\left(10^{6}/\mu L\right)$	Hg (g/dL)	Ht (%)	MCV (fl)	MCHC (%)	Na (mEq/L)	K (mEq/L)
P1	6.64 ^A^	12.03 ^A^	29.83 ^A^	51.68 ^A^	19.97 ^A^	138.83 ^A^	4.44 ^A^
P2	6.57 ^A^	12.15 ^A^	31.08 ^A^	48.41 ^A^	18.83 ^A^	138.67 ^A^	3.96 ^B^

In each column, variable mean values with different capital superscript letters are significantly different between periods (*p* < 0.05).

**Table 3:** Means of white blood cells variables in P1 and P2.

Treatments	Variables						
	Leu (Cel/mm^3^)	Nsg (Cel/mm^3^)	Lym (Cel/mm^3^)	Eos (Cel/mm^3^)	Bas (Cel/mm^3^)	Mon (Cel/mm^3^)	Nrd (Cel/mm^3^)
P1	11667 ^A^	4272.50 ^A^	6780.33 ^A^	438.92 ^A^	46.33 ^A^	128.58 ^A^	25.00 ^A^
P2	11825 ^A^	5238.13 ^A^	6154.96 ^A^	295.08 ^A^	10.66 ^A^	88.00 ^A^	38.17 ^A^

In each column, variable mean values with different capital superscript letters are significantly different between periods (*p* < 0.05).
